# Interplay among pain intensity, sleep disturbance and emotion in patients with non-specific low back pain

**DOI:** 10.7717/peerj.3282

**Published:** 2017-05-16

**Authors:** Shilabant Sen Sribastav, He Peiheng, Long Jun, Li Zemin, Wei Fuxin, Wang Jianru, Liu Hui, Wang Hua, Zheng Zhaomin

**Affiliations:** 1 Department of Orthopaedics, First Affiliated Hospital of Sun Yat-sen University, Guangzhou, P.R. China; 2 Pain Research Center and Department of Physiology, Zhongshan Medical School of Sun Yat-Sen University, Guangzhou, P.R. China

**Keywords:** Sleep disorders, Anxiety, Depression, Low back pain

## Abstract

**Background:**

Low back pain (LBP) is the most common problem worldwide. There are several negative consequences of LBP, such as sleep disorders, work leave, disability, depression, anxiety, and poor quality of life. In this study, we designed to evaluate the prevalence of sleep disturbance in patients with non-specific LBP(NSLBP), and cross-correlation among sleep disorder, anxiety, depression and pain intensity in patients with NSLBP.

**Aim:**

In this study, we designed to evaluate the prevalence of sleep disturbance in patients with NSLBP, and cross-correlation among sleep disorder, anxiety, depression and pain intensity in patients with NSLBP.

**Methods:**

A cross-sectional self-assessment questionnaire survey was carried out in an outpatient clinic. Anonymous assessments were used to characterize the presence of NSLBP, PSQI, VAS, SF-36 form, ODI, BAI and BDI. Cross-correlation among the severity of NSLBP and sleep disturbance, anxiety, depression and life quality were evaluated.

**Results:**

Patients with NSLBP have a higher incidence of sleep disorder, anxiety and depression, and higher ODI scores than healthy people without LPB (*P* < 0.01). NSLBP patients with sleep disorders have more severe anxiety, depression, an increased VAS score and poor daily living (*P* < 0.05). NSLBP patients with anxiety have declined sleep quality, poor daily living, decreased work and social skills, and increased LBP severity (*P* < 0.05). NSLBP patients with depression have declined sleep quality, poor daily living, decreased work and social skills (*P* < 0.05). Significant associations were found between the severity of NSLBP and sleep disorders, anxiety and ODI scores.

**Conclusion:**

Psychological and social factors play an important role in the development of NSLBP. NSLBP leads to sleep disorders, which decrease the sleep quality and increase the unpleasant emotions and memories in return; these can exacerbate the severity of LBP, with the cycle repeating to form a vicious circle.

## Introduction

Low back pain (LBP) is defined as mild to severe pain in the area of the lumbar, lumbo-sacral or sacroiliac regions (Vrbanic, 2011). It is a major global health and socio-economic problem and the most common musculoskeletal disease, with the reported lifetime prevalence ranging from 40% to 85% (Fujii & Matsudaira, 2013), annual prevalence from 25% to 60% (Livshits et al., 2011), and point prevalence 11.9% ± 2.0% (Hoy et al., 2012). The economic burden of LBP is huge, with direct healthcare expenditures of over $90 billion annually in the United States (Borczuk, 2013). A total of 85% of the LBP cases are described as nonspecific (Maatta et al., 2015). The most troubling aspect of LBP is functional disability (Hush et al., 2009), associated with increased health care spending (Ferreira et al., 2010), decreased daily physical function, impaired psychological well-being, and poor quality of life (Sezgin et al., 2014).

Sleep is essential to keep the normal status of emotional, mental and physical health. Sleep disturbance lead to decreased work ability, increased sick leave, and a higher injury rate (Hillman, Murphy & Pezzullo, 2006). Several studies have confirmed that patients with pain have sleep disturbance (Azevedo et al., 2011; Van de Water, Eadie & Hurley, 2011). The prevalence of sleep disturbance in patients with chronic LBP is more than 50% (Bahouq et al., 2013). The pain has been reported to have a bidirectional relationship with sleep; pain hinders sleep while sleep disturbance may decrease pain thresholds and the mental capacity to manage pain (Schuh-Hofer et al., 2013). However, the reason is still unclear on whether poor sleep quality makes an individual vulnerable to LBP, or if sleep disturbance is caused by LBP. Therefore, an effort should be made to detect the association of pain and sleep disturbance in order to improve the management of LBP.

Pain and sleep disturbance can impair patients’ life quality, including physical activity, social integration and emotions (Lavigne et al., 2011). Depression has been reported to occur in patients with chronic pain and sleep disturbance (Campbell et al., 2013), which can also influence sleep quality (Spira et al., 2008), but their relationship is poorly understood.

In this study, we designed to evaluate the prevalence of sleep disturbance in patients with NSLBP compared with 112 age- and sex-matched healthy people without pain; then, cross-correlation among NSLBP, sleep disorder, anxiety and depression was analyzed. The results can provide a theoretical basis for the prevention and treatment of NSLBP. The questionnaires in this study are PSQI, SF-36, ODI, BAI and BDI.

## Material and Methods

Patients with non-specific LBP were enrolled from January 2014 to January 2016 in an orthopedic clinic of the First Affiliated Hospital of Sun Yat-sen University and Wenming Hospital; no limitation was set in the aspects of gender, age, height, weight, occupation, educational level and birth place. The chief compliance was LBP, which lasts more than one month. All patients signed informed consent forms after obtaining approval from the Ethics Committee of the First Affiliated Hospital of Sun Yat-sen University. Patients had a certain education level and can understand questionnaire survey. All patients completed questionnaires and lumbar MRI were taken to find out organic diseases (Norton et al., 2016). The questionnaire focuses on patient’s general information, body weight, education, occupation, exercise, smoking and drinking habit.

The exclusion criteria were: individuals whose reported age was <18 years or >65 years; LBP attributed to spine fracture, spine inflammation, spinal tumor, spinal tuberculosis, disc herniation, spinal stenosis, spondylolisthesis, aneurysm or lithiasis; individuals who have mental disorders, history of cancer or severe chronic physical disorders (e.g., hypertension, diabetes, coronary heart disease, chronic kidney disease, bronchitis, asthma, etc.) (Norton et al., 2016).

For the control group without LBP, the control age- and gender- matched asymptomatic subjects was enrolled from healthy volunteers without LBP, with the exclusion criteria of history of malignant tumors, psychiatric diseases, chronic systemic diseases.

### Survey assessment

Participants were evaluated by questionnaires including PSQI, VAS, SF-36, ODI, BAI and BDI.

PSQI is the 24-item survey that evaluates sleep quality, including subjective sleep quality, sleep latency, sleep duration, sleep efficiency, sleep disturbance, use of sleep medication and daytime dysfunction (Alsaadi et al., 2014). One of seven gradual statements (0-3 points) per item to be selected to sum up total score (0–21). Participants completed the PSQI with regard to sleep in the past month.

Pain intensity of patients with NSLBP were assessed by VAS score, with 0  = no pain and 10  = extreme pain, which is widely used to measure pain with good validity and reliability (Eichen et al., 2014).

SF-36 is the 36-item generic quality of life questionnaire. It has an eight scale profile of functional health and well being, including physical function, role function physical, body pain, general health perceptions, vitality, social functioning, role function emotional and mental health. The total score is 0 to 100, with 100 indicating the best possible score. The SF-36 has been recommended for comprehensively measuring the health and quality of life in participants with or without LBP (Hirsch et al., 2014).

ODI has six gradual statements per item and a total score 0–50, with 0–20% of total score indicating minimal disability, 21–40% moderate disability, 41–60% severe disability, 61–80% crippled, 81–100% total incapacitation. The ODI has been reported to have good validity, reliability and responsiveness in people with LBP (Vianin, 2008).

BAI is a 21-item self report questionnaire designed to evaluate anxiety symptoms (Bryan et al., 2015). One of 21 gradual statements (0–3 points) per item is selected to sum up the total score (0–63), with 0 = no anxiety and 63 = severe anxiety.

BDI is the standard questionnaire for evaluation of cognitive, affective and somatic symptoms of depression (Ellegaard & Pedersen, 2012). BDI was validated in several countries, and composed of 21 items that address the cognitive, affective, behavioral and somatic components of depression.

### Statistical analysis

Statistical analysis were conducted using SPSS version 18 and SAS V9.3. The demographic data including age, sex, education, occupation, exercise, smoking, and drinking were evaluated between the two groups. Characteristics of study participants were summarized by means and SDs for continuous variables, by frequencies and percentages for categorical variables. The pain intensity, quality of daily life, dysfunctional and psychosocial factors were compared and analyzed between these two groups. For continuous variable, if the variable was normally distributed and had constant variances, *t*-test ANOVA would be conducted for comparisons tests; for categorical variable, the difference would be tested with the chi-square test. Sleep disturbance, anxiety, depression and other items of psychosocial and emotional relevance were compared with the severity of LBP. Spearman correlation analysis was done to identify the correlation among the PSQI, VAS, SF-36, ODI, BAI and BDI. Factors found to be significant on univariate analysis were included in a multivariate binary logistic regression analysis.

**Figure 1:**
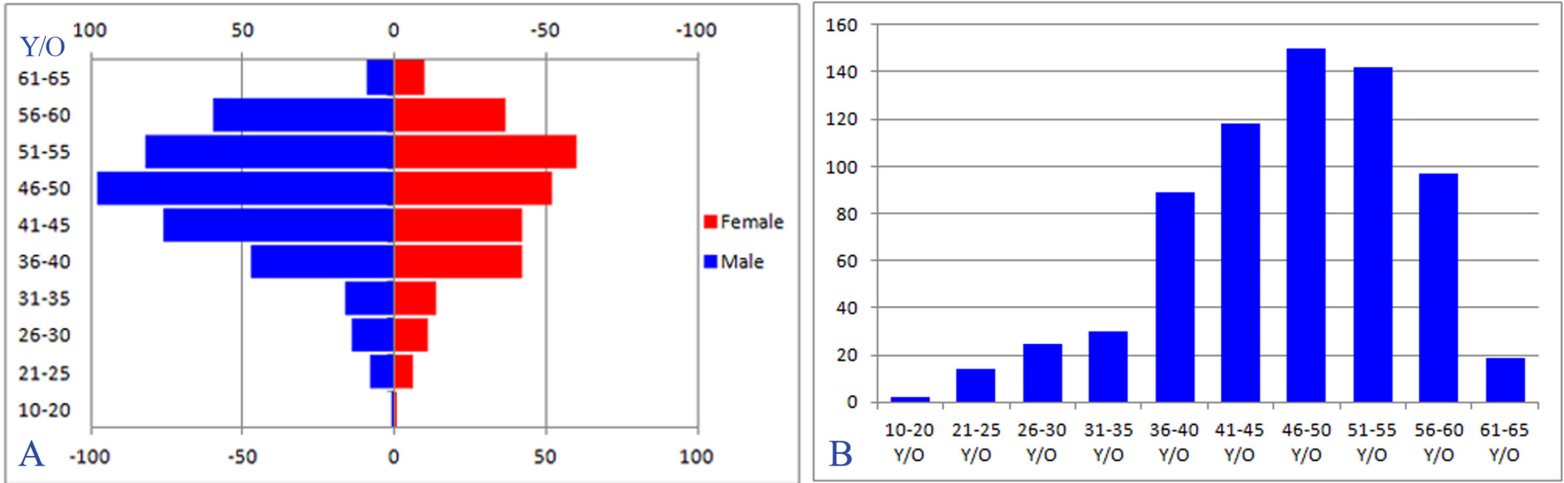
(A/B): Age structure distribution map showed the age of the NSLBP occurrence is mainly concentrated in 31–55 years.

**Table 1:** Demographic data of participants.

Characteristic	Sub-group	LBP patients (*n* = 555)	Healthy control (*n* = 112)	*P* Value	
Gender (n)	Male	325 (58.6%)	68 (60.7%)	χ^2^ = 0.055	*P* = 0.831
	Female	230 (41.4%)	44 (39.3%)		
Age (years)		45.37 ± 9.23	42.13 ± 10.39		*P* = 0.145
Height (meter)		1.59 ± 0.11	1.59 ± 0.12		*P* = 0.531
Weight (Kg)		63.36 ± 11.41	62.18 ± 11.98		*P* = 0.037
Body mass index		25.50 ± 6.24	24.64 ± 4.98		*P* = 0.128
	Normal	310(55.8%)	45(40.2%)	χ^2^ = 74.96	*P* < 0.01
	Over-weight	63(11.4%)	50(44.6%)		
	Obese	182(32.8%)	17(15.2%)		
Smoking (n)	Yes	71 (12.8%)	16 (14.3%)	χ^2^ = 0.183	*P* = 0.669
	No	484 (87.2%)	96 (85.7%)		
Drinking (n)	Yes	103 (18.6%)	20 (17.9%)	χ^2^ = 0.030	*P* = 0.861
	No	452 (81.4%)	92 (82.1%)		
Coffee drinking (n)	Yes	102 (18.4%)	14 (12.5%)	χ^2^ = 2.242	*P* = 0.134
	No	453 (81.6%)	98 (87.5%)		
ODI score		31.74 ± 13.65	7.36 ± 3.32		*P* < 0.01
BAI score (n)				χ^2^ = 32.43	*P* < 0.01
	No anxiety	277 (49.9%)	87 (77.7%)		
	Mild anxiety	163 (29.4%)	21 (18.7%)		
	Moderate anxiety	105 (18.9%)	4 (3.6%)		
	Severe anxiety	10 (1.8%)	0 (0%)		
BDI score (n)				χ^2^ = 31.68	*P* < 0.01
	Normal	176 (31.7%)	65 (58.0%)		
	Mild depression	314 (56.6%)	45 (40.2%)		
	Moderate depression	63 (11.4%)	2 (1.8%)		
	Severe depression	2 (0.3%)	0 (0%)		
PSQI score (n)					
	Subjective sleep quality	1.28 ± 0.66	0.92 ± 0.63		*P* < 0.01
	Sleep latency	1.03 ± 0.77	0.76 ± 0.83		*P* < 0.01
	Sleep duration	0.97 ± 0.60	0.48 ± 0.64		*P* < 0.01
	Sleep efficiency	0.45 ± 0.53	0.27 ± 0.46		*P* < 0.01
	Sleep disturbance	1.19 ± 0.62	0.82 ± 0.60		*P* < 0.01
	Use of sleep medication	0.16 ± 0.38	0.04 ± 0.21		*P* = 0.03
	Daytime dysfunction	0.78 ± 0.68	0.35 ± 0.55		*P* < 0.01

**Notes.**

Group A: patients with LBP; Group B: healthy control participants. Mean (SD).

* *p* < 0.05.

** *p* < 0.01.

Pittsburgh sleep quality index (PSQI), Visual Analogue Scale (VAS), SF-36 form, Oswestry disability index (ODI), Beck anxiety inventory (BAI) and Beck depression inventory (BDI).

## Results

### Participant’s demographic and clinical characteristics

A total of 555 NSLBP patients were enrolled. There were 325(58.6%) men, 230 (41.4%) women, with mean age 37.74  ± 11.10 years (age distribution in Fig. 1), weight 65.22 ± 11.53 kg, and height 161 ± 11 cm. Mean pain intensity on the VAS was 4.35 ± 1.61. Among NSLBP patients, 71 (12.8%) have a smoking habit, 103 (18.6%) have a drinking habit, ODI score was 31.74 ± 13.65, 278 (50.1%) NSLBP patients have the symptom of anxiety with BAI scores 15.95 ± 8.91, 379 (68.3%) have depression with BDI scores 7.65 ± 4.63, 161 (29.0%) have sleep disorders with PSQI scores of 5.85 ± 2.52 (Table 1).

A total of 112 healthy people without LBP were enrolled. There were 68 (60.7%) men, 44 (39.3%) women, with mean age 36.04 ± 11.55 years, weight 62.18 ± 11.98 kg, and height 159 ± 12 cm. Mean pain intensity on the VAS was 4.35 ± 1.61. 16 (14.3%) have a smoking habit, 20 (17.9%) have a drinking habit, ODI scores were 7.36 ± 3.32, 18 (16.1%) included healthy people have the symptom of anxious, with BAI scores of 7.64 ± 6.09, 13 (11.6%) have depression with BDI scores of 2.28 ± 2.20, and 14 (12.5%) have sleep disorders with PSQI scores of 3.28 ± 2.66 (Table 1).

There was no significant difference in age, sex, height, weight, smoking and drinking habits between the two groups (*p* > 0.05). NSLBP patients have higher ODI scores and more incidences of anxiety and depression than healthy people (*P* < 0.01). NSLBP patients were more likely to have sleep disorders compared with healthy people (*P* < 0.01). Patients were further divided into mild, moderate and severe LBP groups; statistical analysis showed that the level of anxiety increased with the severity of LBP (*P* < 0.05) (Table 2).

**Table 2:** The distribution of PSQI, NRS, SF-36 from, ODI, BAI and BDI in the participants.

Characteristic	Sub-group (Cases)	VAS score		PQSI score		BAI score		BDI score		ODI score	
		Score	*P* value	Score	*P* value	Score	*P* value	Score	*P* value	Score	*P* value
NSLBP	Yes (555)			5.85 ± 2.52	*p* < 0.01	15.95 ± 8.91	*p* < 0.01	7.65 ± 4.63		31.74 ± 13.65	
	No (191)			3.64 ± 2.73		9.90 ± 6.95		4.30 ± 3.41		7.36 ± 3.32	
Severiety of NSLBP	Mild (293)	2.57 ± 0.53	*p* < 0.05	5.46 ± 2.47	*p* < 0.05	14.90 ± 8.07	*p* < 0.05	7.12 ± 4.37	*p* < 0.05	29.36 ± 13.37	*p* < 0.05
	Moderate (112)	4.87 ± 0.83^a^		6.00 ±2.39^a^		15.89 ±9.29^a^		7.86 ± 4.58		32.94 ±13.24^a^	
	Severe (71)	7.01 ±0.12^b^^,^^c^		6.32 ±3.04^b^		19.06 ±8.86^b^^,^^c^		8.23 ± 5.36		33.15 ±15.30^b^	
Sleeping disorders	Yes (161)	4.65 ± 1.70	*t* = 2.747	8.78 ± 0.90	*t* = 26.32	17.31 ± 9.85	*t* = 2.303	8.53 ± 4.89	*t* = 2.863	33.54 ± 14.26	*t* = 1.995
	No (394)	4.23 ± 1.57	*p* = 0.006	4.65 ± 1.91	*p* < 0.01	15.40 ± 8.44	*p* = 0.022	7.30 ± 4.47	*p* = 0.004	31.00 ± 13.34	*p* = 0.046
Anxious	Yes (278)	4.52 ± 1.64	*t* = 2.473	6.39 ± 2.37	*t* = 5.147	23.25 ± 6.16	*t* = 33.82	7.95 ± 4.85	*t* = 1.491	33.18 ± 13.19	*t* = 2.507
	No (277)	4.18 ± 1.57	*p* = 0.014	5.31 ± 2.56	*p* < 0.001	8.63 ± 3.72	*p* < 0.001	7.36 ± 4.39	*p* = 0.137	24.47 ± 14.07	*p* = 0.012
Depression	Yes (379)	4.28 ± 1.69	*t* = 0.686	4.88 ± 2.92	*t* = 6.413	14.72 ± 8.17	*t* = 2.227	2.73 ± 1.14	*t* = 24.79	29.13 ± 13.88	*t* = 3.096
	No (176)	4.39 ± 1.58	*p* = 0.493	6.31 ± 2.18	*p* < 0.001	16.53 ± 9.19	*p* < 0.001	9.94 ± 3.78	*p* = 0.026	32.95 ± 13.38	*p* = 0.02
Smoking	Yes (71)	4.59 ± 1.58	*t* = 1.096	6.25 ± 2.49	*t* = 1.430	18.06 ± 9.43	*t* = 2.137	9.42 ± 4.91	*t* = 3.483	31.38 ± 13.06	*t* = 0.236
	No (484)	4.32 ± 1.61	*p* = 0.274	5.79 ± 2.52	*p* = 0.153	15.64 ± 8.79	*p* = 0.033	7.39 ± 4.53	*p* = 0.001	31.79 ± 13.74	*p* = 0.814
Drinking	Yes (103)	4.68 ± 1.48	*t* = 3.173	6.96 ± 2.25	*t* = 5.088	17.96 ± 9.22	*t* = 2.547	9.01 ± 4.53	*t* = 3.349	34.42 ± 12.70	*t* = 2.225
	No (452)	4.21 ± 1.65	*p* = 0.02	5.38 ± 2.49	*p* < 0.01	15.49 ± 8.78	*p* = 0.011	7.34 ± 4.59	*p* = 0.01	31.12 ± 13.79	*p* = 0.027
Coffee drinking	Yes (102)	4.17 ± 1.70	*t* = 1.292	5.62 ± 2.72	*t* = 1.047	16.12 ± 9.21	*t* = 0.206	7.83 ± 4.58	*t* = 0.433	30.19 ± 12.94	*t* = 1.263
	No (453)	4.39 ± 1.59	*p* = 0.197	5.91 ± 2.47	*p* = 0.295	15.92 ± 8.85	*p* = 0.837	7.61 ± 4.64	*p* = 0.665	32.08 ± 13.79	*p* = 0.207
Weight	Normal (310)	4.34 ± 1.67	*p* > 0.05	5.50 ± 2.53	*p* < 0.05	15.73 ± 9.05	*p* > 0.05	7.48 ± 4.46	*p* > 0.05	30.10 ± 13.28	*p* < 0.05
	Over weight (63)	4.37 ± 1.38		6.71 ±2.39^d^		16.40 ± 8.34		7.79 ± 4.55		35.90 ±14.02^d^	
	Obese (182)	4.38 ± 1.60		6.16 ±2.46^e^		16.18 ± 8.90		7.91 ± 4.94		33.09 ±13.76^e^	

**Notes.**

a Represent there is statistical difference between patients with “mild NSLBP” and “moderate NSLBP”.

b Represent there is statistical difference between patients with “mild NSLBP” and “severe NSLBP”.

c Represent there is statistical difference between patients with “moderate NSLBP” and “severe NSLBP”.

d Represent there is statistical difference between patients with “normal weight” and “over weight”.

e Represent there is statistical difference between patients with “normal weight” and “obese”.

Patients were divided into two groups according to the presence of sleep disorders, anxiety or depressive symptoms. Statistical analysis showed that NSLBP patients with sleep disorders have more severe anxiety, depression, increased VAS score and poor daily living (*P* < 0.05). NSLBP patients with anxiety have declined sleep quality, poor daily living, decreased work and social skills, increased LBP severity (*P* < 0.05). NSLBP patients with depression have declined sleep quality, poor daily living, decreased work and social skills (*P* < 0.05) (Table 2).

We then analyzed the relationship of smoking, drinking habits and body mass index on anxiety, depression, quality of sleep and severity of LBP. Results confirmed that smoking habits can affect a patient’s anxiety and depression mood in NSLBP patients, resulting in the increased incidence of anxiety and depression. Drinking habits can lead to increased incidence of anxiety and depression, decreased quality of sleep, poor daily lives, declined work and social skills (*P* < 0.05). Body mass index in NSLBP patients can affect daily lives, work and social skills, but there was no significant correlation between the degree of obesity and patients’ anxiety, depression, daily lives and VAS score (Table 2).

To further understand the correlation between the severity of NSLBP with sleep quality, mood and activities of daily living, Spearman correlation analysis was done. Results showed that VAS score was significantly correlated with PSQI, BDI, BAI and ODI (*r* = 0.093, *p* = 0.029; *r* = 0.096, *p* = 0.024; *r* = 0.128, *p* = 0.003; *r* = 0.126, *p* = 0.003, respectively). PSQI was significantly correlated with BDI, BAI and ODI (*r* = 0.317, *p* < 0.01; *r* = 0.276, *p* < 0.01; *r* = 0.330, *p* < 0.01, respectively). BDI was significantly correlated with BAI and ODI (*r* = 0.136, *p* = 0.001; *r* = 0.122, *p* = 0.004, respectively). BAI and ODI were also significantly correlated (*r* = 0.149, *p* < 0.01) (Table 3).

In the multiple logistic regression model, all predictive variables were significant after controlling for age, gender, and other variables. The effect of NSLBP VAS score, BAI and BDI on sleep quality PSQI was analyzed, results showed that patients with VAS > 7 were associated with higher PSQI score(OR: 2.248, 95% CI [1.235–4.093]) in comparison to patients with VAS 0–3. Patients with moderate anxiety (BAI 26–35) were associated with higher PSQI score (OR: 2.253, 95% CI [1.357–3.741]) compared with patients without anxiety (Table 4). Next, we evaluated the effects of PSQI, BAI and BDI on VAS score. Results showed sleep quality PSQI scores were strongly correlated with the severity of NSLBP (OR: 1.596, 95 % CI [1.108–2.299]). Patients with moderate anxiety (BAI 26-35) were associated with higher VAS scores (OR: 1.642, 95 % CI [1.024–2.632]) (Table 5). We then evaluted the effect of PSQI, VAS scores and BDI on anxiety BAI scores; results showed sleep quality PSQI score was strongly correlated with the severity of anxious symptom(OR: 0.919, 95% CI [0.859–0.982]) (Table 6). Finally, we evaluted the effect of PSQI, VAS score and BAI on depression BDI scores; results showed no significant correlation (Table 7).

**Table 3:** The relationship among the severity of LBP and the quality of sleep, and the emotion in the patients with NSLBP by Pearson correlation (bilateral).

	VAS	PSQI	BDI	BAI	ODI
VAS	1	*r* = 0.093, *p* = 0.029	*r* = 0.096, *p* = 0.024	*r* = 0.128, *p* = 0.003	*r* = 0.126, *p* = 0.003
PSQI	*r* = 0.093, *p* = 0.029	1	*r* = 0.317, *p* < 0.01	*r* = 0.276, *p* < 0.01	*r* = 0.330, *p* < 0.01
BDI	*r* = 0.096, *p* = 0.024	*r* = 0.317, *p* < 0.01	1	*r* = 0.136, *p* = 0.001	*r* = 0.122, *p* = 0.004
BAI	*r* = 0.128, *p* = 0.003	*r* = 0.276, *p* < 0.01	*r* = 0.136, *p* = 0.001	1	*r* = 0.149, *p* < 0.01
ODI	*r* = 0.126, *p* = 0.003	*r* = 0.330, *p* < 0.01	*r* = 0.122, *p* = 0.004	*r* = 0.149, *p* < 0.01	1

**Table 4:** Adjusted association between PSQI and independent variables in the multiple logistic regression model.

Characteristic	Regression coefficients	Standard error	*P*	*OR*	OR _95%*CI*_	
					Lower limit	Upper limit
Moderate NSLBP	−0.2911	0.2187	0.1831	1.338	0.872	2.054
Severe NSLBP	−0.8101	0.3056	0.0080	2.248	1.235	4.093
Mild depression	−0.2152	0.2196	0.3271	1.240	0.806	1.907
Moderate depression	−0.5267	0.3248	0.1048	1.693	0.896	3.200
Severe depression	−0.4829	1.4887	0.7457	1.621	0.088	29.985
Mild anxiety	−0.2024	0.2210	0.3599	1.224	0.794	1.888
Moderate anxiety	−0.8125	0.2587	0.0017	2.253	1.357	3.741
Severe anxiety	1.3847	1.0837	0.2013	0.250	0.030	2.094

**Notes.**

The severity of NSLBP was divided into three groups: mild NSLBP (VAS 0–3), moderate NSLBP (VAS 4–6), severe NSLBP (VAS > 7). The severity of anxious symptom was also divided into three groups: mild anxiety (BAI = 15–25), moderate anxiety (BAI = 26–35), severe anxiety (BAI > 36). The severity of depression symptom was also divided into three groups, mild depression (BDI = 5–13), moderate depression (BDI = 14–20), severe depression (BDI > 21).

**Table 5:** Adjusted association between PSQI and independent variables in the multiple logistic regression model.

Characteristic	Regression coefficients	Standard error	*P*	*OR*	OR_95%*CI*_	
					Lower limit	Upper limit
PSQI(two-category)	−0.4675	0.1862	0.0121	1.596	1.108	2.299
Mild depression	0.0170	0.1841	0.9263	1.017	0.709	1.459
Moderate depression	0.3497	0.2907	0.2289	1.419	0.803	2.508
Severe depression	2.1709	1.4569	0.1362	8.766	0.504	152.377
Mild anxiety	0.2453	0.1873	0.1903	1.278	0.885	1.845
Moderate anxiety	0.4958	0.2408	0.0395	1.642	1.024	2.632
Severe anxiety	0.8579	0.6348	0.1766	2.358	0.680	8.183

**Notes.**

The severity of NSLBP was divided into three groups: mild NSLBP (VAS 0–3), moderate NSLBP (VAS 4–6), severe NSLBP (VAS > 7). The severity of anxious symptom was also divided into three groups: mild anxiety (BAI = 15–25), moderate anxiety (BAI = 26–35), severe anxiety (BAI > 36). The severity of depression symptom was also divided into three groups: mild depression (BDI = 5–13), moderate depression (BDI = 14–20), severe depression (BDI > 21).

**Table 6:** Adjusted association between PSQI and independent variables in the multiple logistic regression model.

Characteristic	Regression coefficients	Standard error	*P*	*OR*	OR_95%*CI*_	
					Lower limit	Upper limit
PSQI	−0.0850	0.0340	0.0126	0.919	0.859	0.982
Moderate NSLBP	0.2261	0.1783	0.2048	1.254	0.884	1.778
Severe NSLBP	−0.0869	0.2682	0.7459	0.917	0.542	1.551
Mild depression	0.1252	0.1847	0.4979	1.133	0.789	1.628
Moderate depression	0.1412	0.2909	0.6275	1.152	0.651	2.037
Severe depression	1.3460	1.3481	0.3181	3.842	0.274	53.958

**Notes.**

The severity of NSLBP was divided into three groups: mild NSLBP (VAS 0–3), moderate NSLBP (VAS 4–6), severe NSLBP (VAS > 7). The severity of anxious symptom was also divided into three groups, mild anxiety (BAI = 15–25), moderate anxiety (BAI = 26–35), severe anxiety (BAI > 36). The severity of depression symptom was also divided into three groups: mild depression (BDI = 5–13), moderate depression (BDI = 14–20), severe depression (BDI > 21).

**Table 7:** Adjusted association between BDI and independent variables in the multiple logistic regression model.

Characteristic	Regression coefficients	Standard error	*P*	*OR*	OR_95%*CI*_	
					Lower limit	Upper limit
PSQI	0.0426	0.0350	0.2233	1.043	0.974	1.118
Moderate NSLBP	0.0485	0.1858	0.9792	1.005	0.698	1.446
Severe NSLBP	0.4121	0.2717	0.1294	1.510	0.886	2.572
Mild anxiety	0.1801	0.1913	0.3465	1.197	0.823	1.742
Moderate anxiety	0.2192	0.2492	0.3791	1.245	0.764	2.029
Severe anxiety	−0.0766	0.6482	0.9060	0.926	0.260	3.300

**Notes.**

The severity of NSLBP was divided into three groups: mild NSLBP (VAS 0–3), moderate NSLBP (VAS 4–6), severe NSLBP (VAS > 7). The severity of anxious symptom was also divided into three groups: mild anxiety (BAI = 15–25), moderate anxiety (BAI = 26–35), severe anxiety (BAI > 36). The severity of depression symptom was also divided into three groups, mild depression (BDI = 5–13), moderate depression (BDI = 14–20), severe depression (BDI > 21).

## Discussion

In this study we investigated the association between pain intensity and sleep quality in non-specific LBP patients, together with the relationship between pain intensity, patients’ life quality, ODI score and emotions. Most of the previous studies on LBP and sleep disturbance focused on patients with persistent pain (Marty et al., 2008). In this study, we included patients with acute and chronic LBP, and we demonstrated that 29.0% of NSLBP patients and 12.5% included healthy people have sleep disturbance, healthy people usually have slight sleep disturbance in older age, while NSLBP patients have more severe sleep disturbance of all age. The prevalence of sleep disturbance in our study is lower than previous study (50–55%) (O’Donoghue et al., 2009).

### Sleep and pain intensity in NSLBP patients

In this study, we found pain intensity is associated with the influence of sleep disturbance, woth severe NSLBP patients having more sleep disturbance. This is similar to previous study, which demonstrated that pain intensity was the most likely influence factor in LBP patients to be associated with sleep disturbance (Alsaadi et al., 2011). Several studies also confirmed significant relationship between sleep disturbance and pain intensity in patients with musculoskeletal pain (Vitiello et al., 2009). However, Lewandowski et al. (2010) reported day-time pain doesn’t associated with objectively and subjectively assessed disturbed sleep. Tang et al. (2012) determined that pain didn’t affect the subsequent night’s sleep in patients with chronic pain and insomnia. The relationship of pain and sleep disturbance is conflicting, which may depend on the study design. It was said that some factors may confuse the results, such as chronic patients and sleep disturbance patients have higher BMI compared with control (Vorona et al., 2005). Smoking also has been described strongly associated with sleep disturbance and LBP (Van de Water, Eadie & Hurley, 2011). Therefore, we used multivariate regression to rule out the influence of these baseline factors, such as BMI, drinking and smoking. Results showed pain intensity and negative moods such as anxiety and depression have been strongly associated with sleep disturbance and pain (Wang et al., 2010).

Pain has been reported to have bidirectional relationship with sleep; pain hinders sleep, while sleep disturbance also decreases pain thresholds and the mental capacity to manage pain (Schuh-Hofer et al., 2013). Austad et al. (2016) demonstrated sleep disturbance was moderately correlated to pain, fatigue, physical function, and utility in patients with rheumatoid arthritis. Therefore, we designed to evaluate the effect of sleep disturbance on the severity of pain intensity in NSLBP patients. Results showed patients with sleep disturbance have higher pain intensity compared to LBP patients without sleep disturbance, which is similar with previous study (O’Donoghue et al., 2009). The presence of disturbed sleep increase fatigue, daytime sleepiness and low mood, which may lead to negative impact individual such as weakening memory, work barrier, stress in daily life, poor life quality and more severe pain (Sezgin et al., 2014).

### Sleep and depression in NSLBP patients

Studies have indicated that LBP was associated with patients’ emotional status (Ramond et al., 2011). Polatin et al. (1993) reported 59% of the investigated LBP patients has psychiatric illnesses, the most frequently mentioned psychiatric illness are depression and anxiety. The prevalence of anxiety and depression were reported 48.57% and 55% respectively (Sagheer, Khan & Sharif, 2013) in chronic LBP patients. The current study also demonstrated significantly higher incidence of depression (68.3% *vs* 11.6%) and anxiety (50.1 *vs* 16.1%) in NSLBP patients compared with healthy people. Arnstein et al. (1999) demonstrated that severe pain is an important factor for depression and disability. Depression also has a bidirectional relationship with pain. Bair et al. (2003) demonstrated that depression is associated with more pain sites, greater pain intensity, longer duration of pain, and increased risk of poor treatment response. Depression produces substantial disability and decrements in health-related quality of life (Bair et al., 2008). Hung, Liu & Fu (2015) found depression was the most powerful factor associated with disability in LBP patients.

In relationship of sleep quality and emotional status, significant correlation between depression, anxiety, and sleep disturbance was well established (Guo et al., 2014), and insufficient sleep may result in depression (Xu et al., 2012). Longitudinal studies have identified insomnia as a risk factor for new-onset or recurrent depression, and this association has been identified in young, middle-aged and older adults (Franzen & Buysse, 2008). In clinical practice, sleep disorder and prolonged use of sleep medication may be early indicators or reversible risk factor for depression (Jaussent et al., 2011). Sleep disturbance was determined to be more prevalent among Chinese adolescents with depressive symptoms (Guo et al., 2014). Gregory et al. (2011) demonstrated sleep deprivation exacerbates fatigue, depression and pain, and sleep disturbance was moderately correlated with anxiety (*r* = 0.39) and depression (*r* = 0.50). This may create a perpetual cycle, where sleep deprivation leads to fatigue and apathy at work, impaired academic and social functioning, reduced levels of motivation, and impaired ability to regulate mood and emotional responses. In addition, depression is identified as the most frequent cause of chronic insomnia in both clinical and epidemiological samples (Tetsunaga et al., 2013). Emery, Wilson & Kowal (2014) found participants with depressive disorder had higher self-reports of pain and disability.

### Sleep and anxiety in NSLBP patients

Anxiety disorders has been reported to be present in up to 60% of patients with chronic pain (Fishbain et al., 1986). Varni et al. (1996) found that the pain intensity has been associated with the severity of anxiety. Bair et al. (2008) found that musculoskeletal pain is much more disabling when depression and anxiety were both present. In addition, anxiety and sleep disturbance frequently co-occur, and this association persists across the lifespan (Spira et al., 2009). Sleep disturbance is commonly observed in individuals with anxiety and related disorders, and recent research suggests that sleep disturbance may predict the development of an anxiety disorder (Batterham, Glozier & Christensen, 2012). Stress is known to be the most common cause of transient insomnia. Anxiety and depression preceded the development of sleep disturbance among young women, whereas anxiety and sleep had a bidirectional association in men and older women (Spira et al., 2009). The bidirectional effects of sleep, anxiety or depression can be seen in many different hypothetical scenarios.

### Effect on life quality

LBP is a disabling disease which restricts life quality, and psychological factors may have a larger impact on disability and quality of life than pain itself (Scholich et al., 2012). Previous studies have investigated the quality of life in LBP patients, and SF-36 is the most widely used HRQL forms to assess health concept in diseased groups as well as general population (Demiral et al., 2006). Soysal, Kara & Arda (2013) suggested the quality of life evaluated by SF-36 has significant impairment among LBP patients. Sleep disturbance, depression and anxiety are major factors affecting the life quality in patients with LBP. Granja et al. (2005) demonstrated that LBP and sleep disturbances were significantly associated with worse HRQoL in all dimensions. The incidence of disability in LBP patients has been reported to be 65%, and the disability assessed by ODI score was higher in LBP patients than healthy people. ODI is a validated tool to assess symptoms severity and functional change in patients with chronic LBP (Herndon, Zoberi & Gardner, 2015). Altug et al. (2015) demonstrated that LBP affects mobility of the patients, LBP patients feel their-selves more depressive. Houde, Cabana & Leonard (2015) reported that significant positive association was found between pain intensity and disability for both young and older individuals, with stronger correlation in the young group. In this study we evaluate the effect of LBP characteristic on patient’s life quality, disability score and emotional property. We found that the disability and life quality decreased with the increased pain, and relationship of disability and life quality was inverse. This is similar to a previous study (Breivik et al., 2006; Harker et al., 2012).

### Strengths and weaknesses of the study

The strength of the present study is that the results are based on the large prospective cohort study. A medical assistant in our research group distributed and helped patients to fill the questionnaire, which can improve the reliability of results. The response rate of our participants was more than 98%. We used a standard definition of non-specific LBP by experts in the field (Norton et al., 2016), MRI and medical history was taken to exclude organic diseases (e.g., spinal tumor, inflammatory, etc.) that could have strong effect on results.

The current study have some limitations. In this cohort study, the accuracy and completeness of the data within the database were dependent on the self-reported questionnaire, which may affect the internal validity of our study. The cross-sectional nature of the analyses limits causal inferences regarding the relationship between depression, anxiety and pain outcomes. Depression and anxiety were assessed with self-report measures rather than in standardized clinical interviews to diagnose specific mental disorders. Findings may not generalize to all racial and ethnic groups, as our samples were mostly southern Chinese. Age distribution may influence the depression scores in low back pain (Calvo-Lobo et al., 2017); further study may make full consideration of these limitations.

### Implications of the study

The identification of interplay among pain intensity, sleep disturbance and emotion in patients with NSLBP has several potential uses. First, there are cross-relationship among pain intensity, sleep disturbance, anxiety and depression; Second, LBP also influenced by the social and psychological status of the patients and their quality of life (Nguyen & Randolph, 2007). Third, we should evaluate the sleep quality and emotion status of the LBP patients during clinical visit, and drug may be needed to treat the sleep disorder, anxiety and depression along with LBP. Fourth, though the importance of sleep and its association with anxiety, depression and pain intensity in LBP patients is gaining attention, much remains to be learned. To more fully elucidate the interplay of sleep, pain intensity and expression of psychological symptoms, cross-sectional and longitudinal research is needed.

## Conclusion

In this study, we evaluate the cross-correlation among sleep disorder, anxiety, depression and pain intensity in patients with NSLBP. Psychological and social factors play an important role in the development of NSLBP. NSLBP lead to sleep disorder, which decrease the sleep quality and increase the unpleasant emotions and memories in return; these can exacerbate the severity of LBP, the cycle can repeating to form a vicious circle.

##  Supplemental Information

10.7717/peerj.3282/supp-1 Data S1 Raw data

## Additional Information and Declarations

### Data Availability

The following information was supplied regarding data availability: The raw data has been supplied as Data S1.

## References

[ref-1] Alsaadi SM, McAuley JH, Hush JM, Lo S, Lin CW, Williams CM, Maher CG (2014). Poor sleep quality is strongly associated with subsequent pain intensity in patients with acute low back pain. Arthritis Rheumatol.

[ref-2] Alsaadi SM, McAuley JH, Hush JM, Maher CG (2011). Prevalence of sleep disturbance in patients with low back pain. European Spine Journal.

[ref-3] Altug F, Kavlak E, Kurtca MP, Unal A, Cavlak U (2015). Comparison of pain intensity, emotional status and disability level in patients with chronic neck and low back pain. Journal of Back and Musculoskeletal Rehabilitation.

[ref-4] Arnstein P, Caudill M, Mandle CL, Norris A, Beasley R (1999). Self efficacy as a mediator of the relationship between pain intensity, disability and depression in chronic pain patients. Pain.

[ref-5] Austad C, Kvien TK, Olsen IC, Uhlig T (2016). Sleep disturbance in patients with rheumatoid arthritis is related to fatigue, disease activity, and other patient-reported outcomes. Scandinavian Jouranl of Rheumatology.

[ref-6] Azevedo E, Manzano GM, Silva A, Martins R, Andersen ML, Tufik S (2011). The effects of total and REM sleep deprivation on laser-evoked potential threshold and pain perception. Pain.

[ref-7] Bahouq H, Allali F, Rkain H, Hmamouchi I, Hajjaj-Hassouni N (2013). Prevalence and severity of insomnia in chronic low back pain patients. Rheumatology International.

[ref-8] Bair MJ, Robinson RL, Katon W, Kroenke K (2003). Depression and pain comorbidity: a literature review. Archives of Internal Medicine.

[ref-9] Bair MJ, Wu J, Damush TM, Sutherl JM, Kroenke K (2008). Association of depression and anxiety alone and in combination with chronic musculoskeletal pain in primary care patients. Psychosomatic Medicine.

[ref-10] Batterham PJ, Glozier N, Christensen H (2012). Sleep disturbance, personality and the onset of depression and anxiety: prospective cohort study. Australian & New Zealand Journal of Psychiatry.

[ref-11] Borczuk P (2013). An evidence-based approach to the evaluation and treatment of low back pain in the emergency department. Emergency Medicine Practice.

[ref-12] Breivik H, Collett B, Ventafridda V, Cohen R, Gallacher D (2006). Survey of chronic pain in Europe: prevalence, impact on daily life, and treatment. European Journal of Pain.

[ref-13] Bryan CJ, Gonzales J, Rudd MD, Bryan AO, Clemans TA, Ray-Sannerud B, Wertenberger E, Leeson B, Heron EA, Morrow CE, Etienne N (2015). Depression mediates the relation of insomnia severity with suicide risk in three clinical samples of USS military personnel. Depression and Anxiety.

[ref-14] Calvo-Lobo C, Vilar Fernandez JM, Becerro-de Bengoa-Vallejo R, Losa-Iglesias ME, Rodriguez-Sanz D, Palomo Lopez P, Lopez Lopez D (2017). Relationship of depression in participants with nonspecific acute or subacute low back pain and no-pain by age distribution. Journal of Pain Research.

[ref-15] Campbell P, Tang N, McBeth J, Lewis M, Main CJ, Croft PR, Morphy H, Dunn KM (2013). The role of sleep problems in the development of depression in those with persistent pain: a prospective cohort study. Sleep.

[ref-16] Demiral Y, Ergor G, Unal B, Semin S, Akvardar Y, Kivircik B, Alptekin K (2006). Normative data and discriminative properties of short form 36 (SF-36) in Turkish urban population. BMC Public Health.

[ref-17] Eichen PM, Achilles N, Konig V, Mosges R, Hellmich M, Himpe B, Kirchner R (2014). Nucleoplasty, a minimally invasive procedure for disc decompression: a systematic review and meta-analysis of published clinical studies. Pain Physician.

[ref-18] Ellegaard H, Pedersen BD (2012). Stress is dominant in patients with depression and chronic low back pain. A qualitative study of psychotherapeutic interventions for patients with non-specific low back pain of 3–12 months’ duration. BMC Musculoskeletal Disorders.

[ref-19] Emery PC, Wilson KG, Kowal J (2014). Major depressive disorder and sleep disturbance in patients with chronic pain. Pain Research and Management.

[ref-20] Ferreira ML, Machado G, Latimer J, Maher C, Ferreira PH, Smeets RJ (2010). Factors defining care-seeking in low back pain—a meta-analysis of population based surveys. European Journal of Pain.

[ref-21] Fishbain DA, Goldberg M, Meagher BR, Steele R, Rosomoff H (1986). Male and female chronic pain patients categorized by DSM-III psychiatric diagnostic criteria. Pain.

[ref-22] Franzen PL, Buysse DJ (2008). Sleep disturbances and depression: risk relationships for subsequent depression and therapeutic implications. Dialogues in Clinical Neuroscience.

[ref-23] Fujii T, Matsudaira K (2013). Prevalence of low back pain and factors associated with chronic disabling back pain in Japan. European Spine Journal.

[ref-24] Granja C, Lopes A, Moreira S, Dias C, Costa-Pereira A, Carneiro A (2005). Patients’ recollections of experiences in the intensive care unit may affect their quality of life. Critical Care.

[ref-25] Gregory AM, Buysse DJ, Willis TA, Rijsdijk FV, Maughan B, Rowe R, Cartwright S, Barclay NL, Eley TC (2011). Associations between sleep quality and anxiety and depression symptoms in a sample of young adult twins and siblings. Journal of Psychosomatic Research.

[ref-26] Guo L, Deng J, He Y, Deng X, Huang J, Huang G, Gao X, Lu C (2014). Prevalence and correlates of sleep disturbance and depressive symptoms among Chinese adolescents: a cross-sectional survey study. BMJ Open.

[ref-27] Harker J, Reid KJ, Bekkering GE, Kellen E, Bala MM, Riemsma R, Worthy G, Misso K, Kleijnen J (2012). Epidemiology of chronic pain in denmark and sweden. Pain Research and Treatment.

[ref-28] Herndon CM, Zoberi KS, Gardner BJ (2015). Common questions about chronic low back pain. American Family Physician.

[ref-29] Hillman DR, Murphy AS, Pezzullo L (2006). The economic cost of sleep disorders. Sleep.

[ref-30] Hirsch O, Strauch K, Held H, Redaelli M, Chenot JF, Leonhardt C, Keller S, Baum E, Pfingsten M, Hildebrandt J, Basler HD, Kochen MM, Donner-Banzhoff N, Becker A (2014). Low back pain patient subgroups in primary care: pain characteristics, psychosocial determinants, and health care utilization. Clinical Journal of Pain.

[ref-31] Houde F, Cabana F, Leonard G (2015). Does age affect the relationship between pain and disability? a descriptive study in individuals suffering from chronic low back pain. Journal of Geriatric Physical Therapy.

[ref-32] Hoy D, Bain C, Williams G, March L, Brooks P, Blyth F, Woolf A, Vos T, Buchbinder R (2012). A systematic review of the global prevalence of low back pain. Arthtitis and Rheumatism.

[ref-33] Hung CI, Liu CY, Fu TS (2015). Depression: an important factor associated with disability among patients with chronic low back pain. International Journal of Psychiatry in Medicine.

[ref-34] Hush JM, Refshauge K, Sullivan G, De Souza L, Maher CG, McAuley JH (2009). Recovery: what does this mean to patients with low back pain?. Arthtitis and Rheumatism.

[ref-35] Jaussent I, Bouyer J, Ancelin ML, Akbaraly T, Peres K, Ritchie K, Besset A, Dauvilliers Y (2011). Insomnia and daytime sleepiness are risk factors for depressive symptoms in the elderly. Sleep.

[ref-36] Lavigne GJ, Nashed A, Manzini C, Carra MC (2011). Does sleep differ among patients with common musculoskeletal pain disorders?. Current Rheumatology Reports.

[ref-37] Lewandowski AS, Palermo TM, De la Motte S, Fu R (2010). Temporal daily associations between pain and sleep in adolescents with chronic pain versus healthy adolescents. Pain.

[ref-38] Livshits G, Popham M, Malkin I, Sambrook PN, Macgregor AJ, Spector T, Williams FM (2011). Lumbar disc degeneration and genetic factors are the main risk factors for low back pain in women: the UK Twin Spine Study. Annals of the Rheumatic Diseases.

[ref-39] Maatta JH, Wadge S, MacGregor A, Karppinen J, Williams FM (2015). Vertebral endplate (Modic) change is an independent risk factor for episodes of severe and disabling low back pain. Spine.

[ref-40] Marty M, Rozenberg S, Duplan B, Thomas P, Duquesnoy B, Allaert F (2008). Quality of sleep in patients with chronic low back pain: a case-control study. European Spine Journal.

[ref-41] Nguyen TH, Randolph DC (2007). Nonspecific low back pain and return to work. American Family Physician.

[ref-42] Norton G, McDonough CM, Cabral HJ, Shwartz M, Burgess Jr JF (2016). Classification of patients with incident non-specific low back pain: implications for research. Spine Journal.

[ref-43] O’Donoghue GM, Fox N, Heneghan C, Hurley DA (2009). Objective and subjective assessment of sleep in chronic low back pain patients compared with healthy age and gender matched controls: a pilot study. BMC Musculoskeletal Disorders.

[ref-44] Polatin PB, Kinney RK, Gatchel RJ, Lillo E, Mayer TG (1993). Psychiatric illness and chronic low-back pain. The mind and the spine—which goes first?. Spine.

[ref-45] Ramond A, Bouton C, Richard I, Roquelaure Y, Baufreton C, Legr E, Huez JF (2011). Psychosocial risk factors for chronic low back pain in primary care—a systematic review. Family Practice.

[ref-46] Sagheer MA, Khan MF, Sharif S (2013). Association between chronic low back pain, anxiety and depression in patients at a tertiary care centre. Journal of the Pakistan Medical Association.

[ref-47] Scholich SL, Hallner D, Wittenberg RH, Hasenbring MI, Rusu AC (2012). The relationship between pain, disability, quality of life and cognitive-behavioural factors in chronic back pain. Disability and Rehabilitation.

[ref-48] Schuh-Hofer S, Wodarski R, Pfau DB, Caspani O, Magerl W, Kennedy JD, Treede RD (2013). One night of total sleep deprivation promotes a state of generalized hyperalgesia: a surrogate pain model to study the relationship of insomnia and pain. Pain.

[ref-49] Sezgin M, Hasanefendioglu EZ, Ali Sungur M, Incel NA, Cimen O, Kanik A, Shin G (2014). Sleep quality in patients with chronic low back pain: a cross-sectional study assesing its relations with pain, functional status and quality of life. Journal of Back and Musculoskeletal Rehabilitation.

[ref-50] Soysal M, Kara B, Arda MN (2013). Assessment of physical activity in patients with chronic low back or neck pain. Turkish Neurosurgery.

[ref-51] Spira AP, Friedman L, Aulakh JS, Lee T, Sheikh JI, Yesavage JA (2008). Subclinical anxiety symptoms, sleep, and daytime dysfunction in older adults with primary insomnia. Journal of Geriatric Psychiatry and Neurology.

[ref-52] Spira AP, Stone K, Beaudreau SA, Ancoli-Israel S, Yaffe K (2009). Anxiety symptoms and objectively measured sleep quality in older women. American Journal of Geriatric Psychiatry.

[ref-53] Tang NK, Goodchild CE, Sanborn AN, Howard J, Salkovskis PM (2012). Deciphering the temporal link between pain and sleep in a heterogeneous chronic pain patient sample: a multilevel daily process study. Sleep.

[ref-54] Tetsunaga T, Misawa H, Tanaka M, Sugimoto Y, Tetsunaga T, Takigawa T, Ozaki T (2013). The clinical manifestations of lumbar disease are correlated with self-rating depression scale scores. Journal of Orthopaedic Science.

[ref-55] Van de Water AT, Eadie J, Hurley DA (2011). Investigation of sleep disturbance in chronic low back pain: an age- and gender-matched case-control study over a 7-night period. Manual Therapy.

[ref-56] Varni JW, Rapoff MA, Waldron SA, Gragg RA, Bernstein BH, Lindsley CB (1996). Chronic pain and emotional distress in children and adolescents. Journal of Developmental and Behavioral Pediatrics.

[ref-57] Vianin M (2008). Psychometric properties and clinical usefulness of the Oswestry Disability Index. Journal of Chiropractic Medicine.

[ref-58] Vitiello MV, Rybarczyk B, Von Korff M, Stepanski EJ (2009). Cognitive behavioral therapy for insomnia improves sleep and decreases pain in older adults with co-morbid insomnia and osteoarthritis. Journal of Clinical Sleep Medicine.

[ref-59] Vorona RD, Winn MP, Babineau TW, Eng BP, Feldman HR, Ware JC (2005). Overweight and obese patients in a primary care population report less sleep than patients with a normal body mass index. Archives of Internal Medicine.

[ref-60] Vrbanic TS (2011). Low back pain—from definition to diagnosis. Reumatizam.

[ref-61] Wang H, Ahrens C, Rief W, Schiltenwolf M (2010). Influence of comorbidity with depression on interdisciplinary therapy: outcomes in patients with chronic low back pain. Arthritis Research & Therapy.

[ref-62] Xu Z, Su H, Zou Y, Chen J, Wu J, Chang W (2012). Sleep quality of Chinese adolescents: distribution and its associated factors. Journal of Paediatrics and Child Health.

